# Essential role for telomerase in chronic myeloid leukemia induced by BCR-ABL in mice

**DOI:** 10.18632/oncotarget.461

**Published:** 2012-03-08

**Authors:** Carolina Vicente-Dueñas, Marcos Barajas-Diego, Isabel Romero-Camarero, Inés González-Herrero, Teresa Flores, Isidro Sánchez-García

**Affiliations:** ^1^ Experimental Therapeutics and Translational Oncology Program, Instituto de Biología Molecular y Celular del Cáncer, CSIC/ Universidad de Salamanca, SALAMANCA, (SPAIN); ^2^ Institute of Biomedical Research of Salamanca (IBSAL); ^3^ Departamento de Anatomía Patológica, Universidad de Salamanca

**Keywords:** cancer, cancer stem cells (CSC), stem cells, mouse models, Telomerase inhibitors, drug discovery

## Abstract

The telomerase protein is constitutively activated in malignant cells from many patients with cancer, including the chronic myeloid leukemia (CML), but whether telomerase is essential for the pathogenesis of this disease is not known. Here, we used telomerase deficient mice to determine the requirement for telomerase in CML induced by BCR-ABL in mouse models of CML. Loss of one telomerase allele or complete deletion of telomerase prevented the development of leukemia induced by BCR-ABL. However, BCR-ABL was expressed and active in telomerase heterozygous and null leukemic hematopoietic stem cells. These results demonstrate that telomerase is essential for oncogene-induced reprogramming of hematopoietic stem cells in CML development and validate telomerase and the genes it regulates as targets for therapy in CML.

## INTRODUCTION

Activation of telomerase is associated with malignancy in many human cancers [1]. Telomerase inhibition was proposed as a potential cancer therapy when telomere shortening was first described in human cells [2]. Telomerase-deficient mice have been generated via deletion of the mouse *terc* gene encoding the RNA component of telomerase [3]. Terc-/- mice show phenotypic abnormalities only after successive generations of terc-/- intrecrosses [4, 5]. Strong evidence has accumulated that short telomeres limit tumor growth. Crosses of terc-/- mice to tumor prone models demonstrate that the short telomere response significantly limits tumor formation [6]. However, in contrast to these findings in differentiated tumor cells, the contribution of telomerase to the biology of cancer stem cells (CSC) has not been previously investigated.

Recently we have shown that the restricted expression of the *BCR-ABL* oncogene, linked to chronic myeloid leukemia (CML) disease, to the hematopoietic stem cell compartment is capable of generating a full-blown tumour with all its differentiated cellular components, showing a hands-off role for BCR-ABL in regulating CML formation [7-12]. *BCR-ABL* oncogene inactivation could not change this tumor reprogramming fate at the CSC level, in agreement with the common occurrence of tumor relapse by which human CML evolves to escape BCR-ABL pharmacological inactivation [13-22]. Thus, it seems important to know how to eradicate and/or prevent this BCR-ABLp210-induced reprogramming of stem cells [10-12]. In order to identify the genes that are associated with BCR-ABLp210-induced reprogramming of stem cells we performed a supervised analysis of the transcriptional profiles of CSCs purified from *Sca1-BCR-ABLp210* mice versus hematopoietic stem cells from control mice. The data identified that *terc* gene was expressed in CSCs in the Sca1-BCR-ABLp210 mice, although it was not differentially regulated in CSCs versus control hematopoietic stem cells [7, 8]. Now, to assess the functional importance of telomerase and telomere status in this Sca1-BCR-ABLp210 model, the *terc* gene knockout was established in these Sca1-BCR-ABLp210 mice, and tumor phenotype assessed in the first generation of telomerase heterozygous and homozygous mice. Remarkably, the data provide evidence that telomerase activity is essential for BCR-ABLp210-induced reprogramming of stem cells. Overall, our results demostrate that telomerase plays a critical role in the pathogenesis of BCR-ABL-CML, and validate telomerase and the genes it regulates as targets for therapy in CML.

## RESULTS AND DISCUSSION

**Figure 1:**
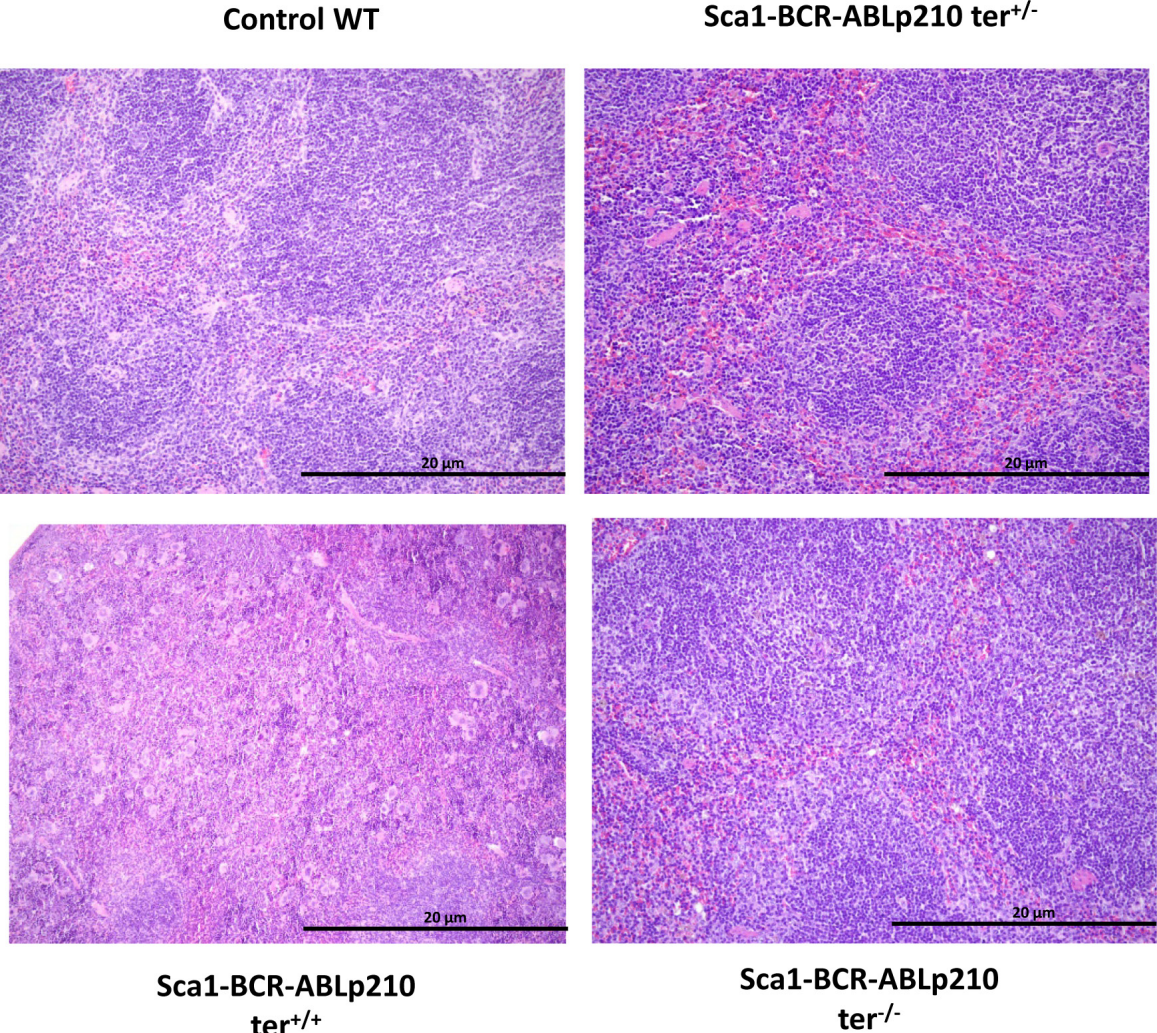
Telomerase is required for CML development in *Sca1-BCR-ABLp21*0 mice Representative histologic appearance of spleen of old *Sca1-BCR-ABLp210 ter^+/−^*, *Sca1-BCR-ABLp210 ter^−/−^*, *Sca1-BCR-ABLp210 ter^+/+^* and control wild-type mice after hematoxylin-eosin staining. Note the organ infiltration by myeloid cells in spleen and the presence of blasts and mature myeloid cells in *Sca1-BCR-ABLp210 ter^+/+^*mice.

**Figure 2:**
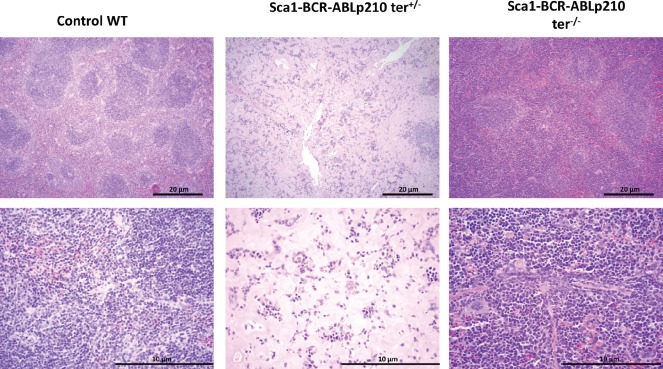
Some *Sca1-BCR-ABLp210 ter^+/−^* and *Sca1-BCR-ABLp210 ter^−/−^* mice develop CML Representative histologic appearance of spleen of old *Sca1-BCR-ABLp210 ter^+/−^ Sca1-BCR-ABLp210 ter^−/−^*, and control wild-type mice after hematoxylin-eosin staining. Note the organ infiltration by myeloid (blasts and mature) cells in spleen.

**Figure 3:**
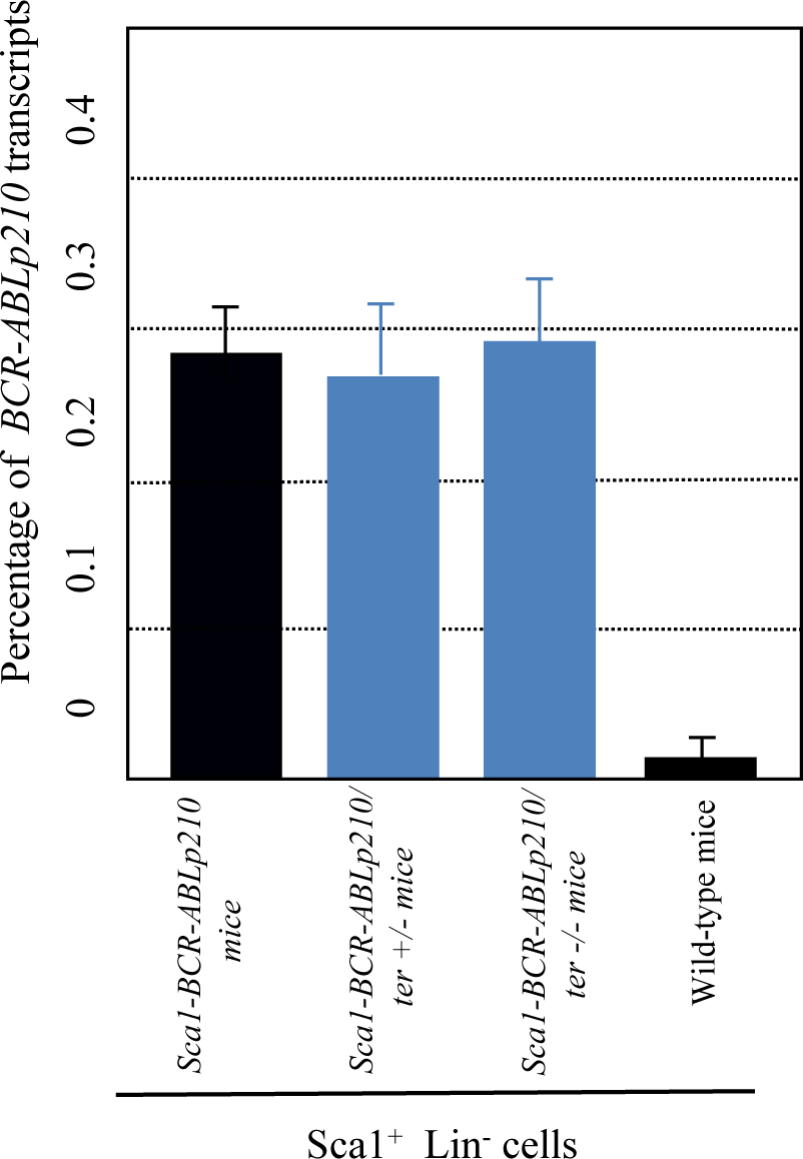
Quantification of *Sca1-CR-ABLp210* expression in Sca1+Lin- cells of *Sca1-BCR-ABLp210 ter^+/−^* and *Sca1-BCR-ABLp210 ter^−/−^* mice Quantification of *BCR-ABLp210* expression in *Sca1-BCRABLp210 ter^+/−^* and *Sca1-BCRABLp210 ter^−/−^* mice by real-time PCR in Sca1^+^Lin^−^ cells. Percentage of *BCRABLp210* transcripts with reference to βactin is shown.

In order to understand the role of telomerase in leukemia stem cell (LSC) generation and maintenance, we have taken advantage of our *Sca1-BCR-ABLp21*0 mouse model of human chronic myeloid leukaemia (CML), a paradigmatic stem-cell disorder [23, 24]. This transgenic mouse was engineered to express the human *BCRABLp210* cDNA under the control of the Sca1 promoter in order to limit and determine the effect of ectopic expression of *BCRABLp210* in hematopoietic stem/progenitor cells [7, 8, 25, 26]. This model not only faithfully recapitulates the human disease but also has been able to anticipate that human CML stem cells survival is Bcr-Abl kinase independent and suggest curative approaches in CML must focus on kinase-independent mechanisms of resistance [7, 27, 28]. This model represents an ideal system to analyze the contributions of telomerase activity deficiency to the LSC biology and malignant progression of CML. To this end, terc-deficient mice were crossed to Sca1-BCR-ABLp210 mice to produce and analyzed cohorts of *Sca1-BCR-ABLp210 ter^+/−^* (n=17) and of *Sca1-BCR-ABLp210 ter^−/−^* (n=17) experimental mice together with *Sca1-BCR-ABLp210 ter^+/+^* (n=21) controls. Mice were monitored clinically and by serial peripheral blood count for evidence of CML for 20 months. As described [7], all *Sca1-BCR-ABLp210 ter^+/+^* mice develop CML (Table I). Surprisingly, when the compound *Sca1-BCR-ABLp210 telomerase*-deficient mice were analysed, CML was not found in the majority of them and the survival of these *Sca1-BCR-ABLp210 ter^+/−^* and *Sca1-BCR-ABLp210 ter^−/−^* mice was significantly increased in comparison with *Sca1-BCR-ABLp210 ter^+/+^* (Table I, Figure 1). Histologic analysis revealed only similar pathology to *Sca1-BCR-ABLp210 ter^+/+^*mice in 4 moribund *Sca1-BCR-ABLp210 ter^+/−^* and 3 moribund *Sca1-BCR-ABLp210 ter^+/+^*mice, respectively (Figure 2). On the contrary, the majority of old *Sca1-BCR-ABLp210 ter^+/−^* and *Sca1-BCR-ABLp210 ter^−/−^* mice do not develop CML as evidenced by the normal spleen sizes and normal leukocyte cellularity in the peripheral blood. The absence of CML disease was further confirmed by histologic analysis that revealed normal spleen in majority old *Sca1-BCR-ABLp210 ter^+/−^* and *Sca1-BCR-ABLp210 ter^−/−^* where we cannot detect the dramatic expansion of progenitors and differentiated myeloid cells that is characteristic of CML (Figure 1). Quantitative RT-PCR of *BCR-ABLp210* messenger mRNA confirmed that BCR-ABL was expressed in telomerase-deficient hematopoietic stem cells (Figure 3). These results indicate that the reduction in telomerase dosage in hematopoietic stem cells does interfere with the development of CML induced by BCR-ABL.

**Table I:** Incidence of CML in Sca1-BCR-ABLp210 ter*^+/−^* and ter*^−/−^* mice

	Sca1-BCR-ABLp210 ter^+/−^	Sca1-BCR-ABLp210 ter^−/−^	Sca1-BCR-ABLp210 ter^+/+^
**Mice with CML**	4	3	21
**Mice without CML**	13	14	0
**Total mice**	n=17	n=17	n=21

Overall, our work demonstrates for the first time that telomerase is required for the generation and maintenance of LSCs. These results demonstrate that telomerase is essential for oncogene-induced reprogramming of hematopoietic stem cells in CML development and validate telomerase and the genes it regulates as targets for therapy in CML. This evidence that telomerase inactivation could modify LSC indicates that the reduction/absence of telomerase does not have an instructive role in the genesis of tumor CML cells, but just a permissive one, preventing cells with damage from being successfully terminally reprogrammed. This indicates that the driving force of the reprogramming process is the *BCR-ABL* oncogene itself. However, targeting of BCR-ABL does not affect CML stem cells. The search for ways to eliminate leukemic stem cells has became a priority due to their resistance to kinase inhibitors and the likely role they play in disease recurrence [29]. A number of strategies have yielded promissing results. Our results add the telomerase pathway as a potential therapeutic target whose inactivation can lead to CML stem cell eradication.

## MATERIALS AND METHODS

### Ethics statement

All animal work has been conducted according to relevant national and international guidelines and it has been approved by the Bioethics Subcommittee of *Consejo Superior de Investigaciones Cientificas* (CSIC).

### Mice

The *Sca1-BCR-ABLp21*0 mice [7] and *terc* gene targeted mice [3] have both been described previously. Southern blot-based genotyping for *Sca1-BCR-ABLp210* [7, 30] and *terc* [3], respectively, were performed as described. Heterozygous *ter^+/−^* mice were bred to *Sca1-BCR-ABLp21*0 mice to generate compound heterozygotes. F1 animals were crossed to obtain null ter*^−/−^* mice heterozygous for *Sca1-BCR-ABLp21*0 mice. Tumor phenotype was assessed in the first generation of telomerase heterozygous and homozygous mice.

### Histological analysis

All mice included in this study were subjected to standard necropsy. All major organs were examined under the dissecting microscope, and samples of each organ were processed into paraffin, sectioned and examined histologically. All tissue samples were taken by the pathologist from homogenous and viable portions of the resected sample and fixed within 2-5 min. of excision. Hematoxylin- and eosin-stained sections of each tissue were reviewed by a single pathologist (T.F.). For comparative studies, age-matched mice were used.

### Real-time PCR quantification

cDNA for use in quantitative PCR studies was synthesized using reverse transcriptase (Access RT-PCR System; Promega, Madison, WI). Two μl of second round amplified RNA was transcribed. Primers and probes used for quantitative PCR are commercially available (TaqMan Assays-on-Demand Gene Expression Products, Applied Biosystems, Foster City, CA). In addition the probes were designed so that genomic DNA would not be detected during the PCR. The sequences of the specific primers and probes were as follow: *BCR*-*ABL^p210^*, sense primer 5'-TTCTGAATGTCATCGTCCACTCA-3', antisense primer 5'-AGATGCTACTGGCCGCTGA-3' and probe 5`-CCACTGGATTTAAGCAGAGTTCAAAAGCCC-3'; c-Abl, sense primer 5'-CACTCTCAGCATCACTAAAGGTGAA-3', antisense primer 5’-CGTTTGGGCTTCACACCATT-3’, and probe 5’-CCGGGTCTTGGGTTATAATCACAATG-3’.

### Analysis and monitoring of disease

Peripheral blood was collected from retro-orbital plexus with a heparinized capillary tube, and total white blood cell and differential counts were performed twice a week. The number of white blood cells was determined with a hemocytometer after lysis of enucleated red blood cells with RCLB lysis buffer (0.15 M NH4Cl; 1 mM KHCO3; 0.1 mM Na2-EDTA, pH 7.4).

### Flow cytometry

Nucleated cells were obtained from total bone marrow (flushing from the long bones), peripheral blood, thymus, liver and spleen. In order to prepare cells for flow cytometry, contaminating red blood cells were lyzed with RCLB lysis buffer and the remaining cells were then washed in PBS with 2% FCS. After staining, all cells were washed once in PBS with 2% FCS containing 2 mg/mL propidium iodide (PI) to allow dead cells to be excluded from both analyses and sorting procedures. Monoclonal antibodies were obtained from Pharmingen and included: Lineage markers (CD45R/B220, for B lineage staining; CD4, CD8 and CD3 for T cell lineage; CD11b and Gr1 for myeloid lineage and TER119 for erythroid lineage) and Sca1 (E13-161.7) for stem cells. Single cell suspensions from the different tissue samples obtained by routine techniques were incubated first with purified anti-mouse CD32/CD16 (Pharmingen) prior to the addition of other antibodies, to block binding via Fc receptors and then with an appropriate dilution of the different antibodies at room temperature or 4 oC, respectively. The samples and the data were analysed in a FACSCalibur using CellQuest software (Becton Dickinson). Specific fluorescence of FITC and PE excited at 488 nm (0.4 W) and 633 nm (30 mW), respectively, as well as known forward and orthogonal light scattering properties of mouse cells were used to establish gates. For each analysis a total of at least 5.000 viable (PI-) cells were assessed.

### Cell purification

For cell sorter separation, bone marrow cells were incubated with anti-Sca1 and anti-lineage markers antibodies (CD3, CD4, CD8, B220, TER119, Gr1, and Mac1). Sca1^+^Lin^−^ and Sca1^−^Lin^+^ cells were isolated and highly purified from the BM of leukemic primary mice or control mice by fluorescence-activated cell sorting (FACS) (FACSVANTAGE; Becton Dickinson). c-kit (CD117) was not used for stem cell isolation as previous studies of human and mouse specimens have described down-regulation of c-kit as a feature of leukemia stem cells [7]. Sorted cells were then re-analyzed for purity with the FACS and determined to be over 98%.

### Statistical analysis

The X^2^ test was used to compare leukemia incidence in *Sca1-BCR-ABLp210 ter^−/−^* mice and *Sca1-BCR-ABLp210 ter^+/−^* versus *Sca1-BCR-ABLp210 and wild-type* mice.
